# Minimising Stress for Patients in the Veterinary Hospital: Why It Is Important and What Can Be Done about It

**DOI:** 10.3390/vetsci4020022

**Published:** 2017-04-13

**Authors:** Janice K. F. Lloyd

**Affiliations:** College of Public Health, Medicine and Veterinary Science, James Cook University, Townsville, QLD 4811, Australia; janice.lloyd@jcu.edu.au

**Keywords:** stress, low stress handling, behaviour modification, behavioural management medications, animal welfare, hospital design

## Abstract

Minimising stress for patients should always be a priority in the veterinary hospital. However, this is often overlooked. While a “no stress” environment is not possible, understanding how to create a “low stress” (sometimes called “fear-free”) environment and how to handle animals in a less stressful manner benefits patients, staff and the hospital alike. Many veterinary practitioners believe creating a low stress environment is too hard and too time consuming, but this need not be the case. With some simple approaches, minimising patient, and hence staff, stress is achievable in all veterinary practices. This article provides a background on why minimising stress is important and outlines some practical steps that can be taken by staff to minimise stress for presenting and hospitalised patients. Useful resources on recognising signs of stress in dogs and cats, handling, restraint, behaviour modification, medications, and hospital design are provided.

## 1. Introduction

The importance of low stress handling in the veterinary hospital or clinic is becoming increasingly recognised around the world (Hewson, 2008 [1]; 2012 [2]; Yin, 2009 [3]; Overall, 2013 [4,5]; Lloyd, 2014 [6]; Tynes, 2014 [7]; AVSAB, 2016 [8]; Harvey, 2016 [9]). Many patients become stressed in the veterinary hospital as they are in unfamiliar surroundings, cannot control or predict what will happen to them, and may be in pain (Hewson, 2008 [1]; 2012 [2]). If prolonged, this stress can have adverse effects on immunity, general health and behaviour (Moberg and Mench, 2000 [10]; Tynes, 2014 [7]), and is of concern to the animal’s owner (client).

One study (Döring, Roscher, Scheipl, Küchenhoff and Erhard, 2009 [11]) that examined the behaviour of clinically healthy dogs visiting a veterinary hospital in Germany found that 78.5% of the dogs studied (*n* = 135) were fearful on the examination table. Fewer than half of the dogs entered the practice calmly and 13.3% had to be dragged or carried into the practice. The Bayer veterinary care usage study (Volk, Felsted, Thomas, and Siren, 2011 [12]) identified that a major reason clients failed to bring their pets to the veterinary hospital was because clients were unwilling to put up with the stress to the animal and to themselves. Veterinarians interviewed in the study stated that many pet owners delayed bringing sick and injured animals to the clinic, with one experienced veterinarian saying, “I’m [now] seeing pets three days sicker.” Many cat owners in the study indicated that their cats acted remote and unfriendly for several days after returning home, which is particularly undesirable in sick or recovering animals.

There is mounting evidence to suggest that routine veterinary care may be contributing to lifelong patient anxiety, starting with the first puppy or kitten physical examination (Overall, 2013 [4,5]; Overall, 2014 [13]). A recent survey of animal welfare experts including veterinarians identified 85 potential factors that could influence dog and cat welfare in relation to veterinary care (Dawson, Dewey, Stone, Guerin, and Niel, 2016 [14]). Factors that impacted on welfare within the hospital environment included: auditory and olfactory stimulation; optimisation of analgesia; patient to patient interactions; separation from the owner and other conspecifics; novel space; physical, visual or temporal separation of patients; and physical restraint. Even a single visit to the veterinary clinic can have a dramatic impact on a pet’s long-term behavioural well-being. A video presentation by Overall (2014 [13]) explains how a frightening experience at the veterinary clinic, for 8–12-week-old animals, whose brain cortices are still developing, can set in motion a chain of neurochemical events that can be detrimental to learning. This is important as many veterinarians mistakenly believe or accept that it is “normal” for dogs and cats to be afraid in the hospital environment. Preliminary results of a study (Godbout and Frank, 2011 [15]) showed that puppies that were fearful in the veterinary environment as young as 2–4 months of age were fearful as young adults (12 months later) in the same contexts. Thus, the veterinary staff’s first impressions of puppy and kitten behaviour are very important, as many puppy behaviours may persist in adulthood and anxious dogs and cats can be identified at a young age (Overall, 2013 [4]).

Without compassionate and respectful handling by the veterinary team, clients may feel the team lacks skills and compassion, or does not understand their pet. Injury may occur to the patients, client and/or veterinary team. In addition to avoiding veterinary visits, clients who want to avoid stress for their pet may choose another practice instead. Thus, the goals of low stress or fear free veterinary practice are to: reduce fear and pain for veterinary patients; reduce anxiety for the client; reinforce the veterinarian-client-pet relationship resulting in better lifelong medical care for the patients; improve efficiency, productivity and job satisfaction for the veterinary team; increase client compliance; enhance timely reporting and early detection of medical and behavioural concerns; and sustain fewer injuries to clients and the veterinary team (Rodan et al., 2011 [16]; Hammerle et al., 2015 [17]). The concept of “low stress handling and restraint” is also relevant to large animals, production animals, exotics etc. If various species of animals in zoos, aquaria and wildlife parks can be trained to participate in their own medical care by presenting body parts for examination/injection, then dogs, cats and other animals should be afforded the opportunity to visit the veterinarian with minimal distress. In recent years, *dvm360*, *Veterinary Economics*, *Veterinary Medicine* and *Firstline* (authorities on veterinary medicine, business, news, etc.) presented a Leadership Challenge devoted to the concept of fear free veterinary care. Veterinary staff can find coverage, analysis, solutions and tools on the fear free concept on-line at http://veterinarynews.dvm360.com/dvm360-leadership-challenge-fear-free.

## 2. The Relationship between Fear and Anxiety

Fear and anxiety are not the same, and it is important that veterinary staff understand the distinction in order to reduce the incidence of fear in the clinic (and in the client’s home). Tynes (2014, [7]) has written a useful article on the physiologic effects of fear: Fear and anxiety share many similar physiologic responses and an animal that experiences fear and anxiety frequently, and is unable to escape from the stimuli, will suffer from stress and its effects. Fear is a normal behaviour. It is an emotional response that occurs when an animal *perceives* that a situation is dangerous, and is adaptive when it helps an animal escape from a situation that is truly dangerous. Anxiety, on the other hand, is *anticipation* of future danger that may be unknown, imagined or real. Chronic anxiety is considered to be an abnormal behaviour and can be a serious welfare concern for an animal in a continual state of anxiety.

Tynes (2014 [7]) also provides some useful definitions of *stress* and *phobia*. Stress is defined as “…any chemical, physical, or emotional force that threatens an organism’s homeostasis”—where the physiologic response (autonomic arousal and stimulation of the hypothalamic–pituitary–adrenal axis and accompanying release of stress hormones) may ultimately result in increased susceptibility to disease secondary to suppression of the immune system. Phobias are “...persistent and excessive fears of certain things or situations that are usually out of proportion to the actual threat that they present”. An animal’s responses to stimuli can range from mild signs of anxiety to extreme panic/catatonia; severely panicked animals can be seriously injured by chewing or charging through doors and windows in an attempt to escape the frightening stimuli. When a stimulus is intensely unpleasant, a single exposure can be enough to induce a fearful response in the future (known as “one-event (trial) learning”). This theory, promoted by American psychologist Edwin Guthrie (Landsberg, Hunthausen and Ackerman, 2013 [18]), supports Tynes (2014 [7]) assumption that some animals are so frightened by their experiences at the veterinary clinic that their responses on subsequent visits could be defined as phobic.

## 3. Recognising Stress in Animals

Non-human animals do not perceive the world as we see it. They see, hear, smell and experience their environment in different ways, yet veterinary hospitals are usually designed with humans in mind and not necessary the patients. Keeping these crucial differences in mind can help greatly when it comes to designing a low-stress environment.

In general, the factors that worsen fear and elicit aggression in dogs also apply to cats. Dogs and cats can experience extreme stress through being separated from their owners and kept in a cage (Dybdall, Strasser, and Katz, 2007 [19]). These animals undergo physiological changes such as increased heart rate and release of cortisol—both of which may be associated with negative feelings such as fear and anxiety (Gregory, 2004 [20]). In addition, stressed animals may not eat or drink adequately, which can delay recovery (Hewson, 2008 [1]). Recognising stress is particularly important in an emergency situation as stressed patients can be difficult to handle, and dog/cat bites and cat scratches are the most common cause of injury to personnel in the veterinary hospital (Jeyaretnam, Jones, and Phillips, 2000 [21]). Recognising signs of stress in the veterinary hospital is also important as the stress response may hinder accurate diagnosis by affecting the results of samples collected (Tynes, 2014 [7]) and lead to increased anaesthetic and sedation risk (AVSAB, 2016 [8]). Furthermore, every time an animal has a bad experience in the veterinary hospital, it is more likely that it will be more fearful and difficult to handle on its next visit due it making an association between the experience and the environment/personnel (classical conditioning). Accepting that dogs and cats are generally afraid in the hospital environment makes it more difficult to recognise the pathologically fearful or anxious animal (Tynes, 2014 [7]).

How an animal behaves in a given situation depends on its genetic predisposition, previous experience and the environment it is in (Steimer, 2002 [22]). Frightened animals may attempt to run away, freeze or become aggressive; pressurising fearful animals in uncertain situations is likely to lead to bites. Taking a few minutes to assess just how fearful a pet is, and altering staff behaviours and the environment can go a long way to encourage patient cooperation. Yin (2009 [3]) has written a useful book with accompanying DVD to help veterinary staff take a more humane approach to companion animal care in the veterinary hospital, with much of this information being freely available on-line at https://drsophiayin.com/.

### 3.1. Signs of Fear in Dogs

**Figure 1:**
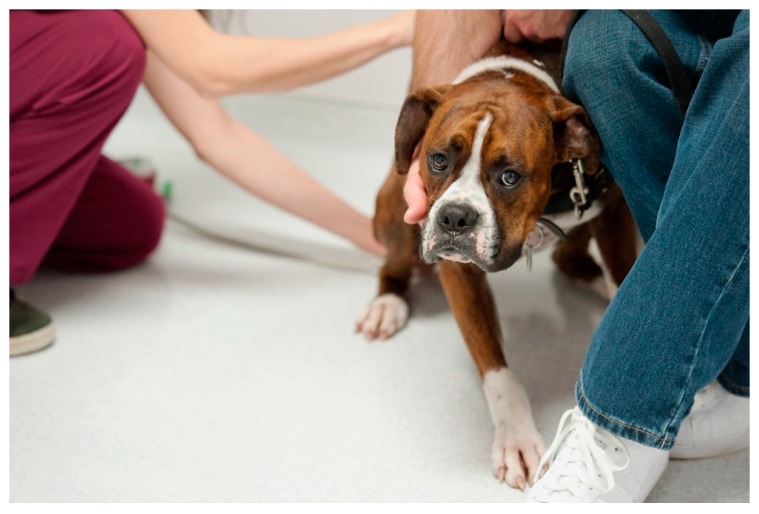
Frightened dog. Leaning away, ears back, whale eye, furrowed brow. Source: https://drsophiayin.com/app/uploads/blogimg/dreamstime_l_14378766.jpg. (Permission received.)

Signs of a fearful dog may include cowering, leaning away with low head/tail and a tense, trembling body. The dog may avert its gaze and show the distinctive “whale eye” (exposed whites of eyes). Ears may be flat against the head and the brow furrowed (Figure 1). Yin (2009 [3]) emphasises the importance of personnel not interpreting fearful postures (designed to keep others from coming closer) as submissive behaviour. If veterinary staff assume the dog is being subordinate, they may reach for the dog and the dog may become fearfully aggressive. More subtle signs of fear/anxiety include scanning the room for danger (hypervigilance), yawning, panting, lip licking, refusing treats, salivating (in the absence of food), looking/acting sleepy (when not tired) or distracted, sniffing, and possible escalation to urination and defecation. A list of non-specific signs of anxiety in dogs can be found in Overall (2013 [23] (p. 47)) or accessed on-line (Overall, 2014 [24]).

### 3.2. Signs of Fear in Cats

**Figure 2:**
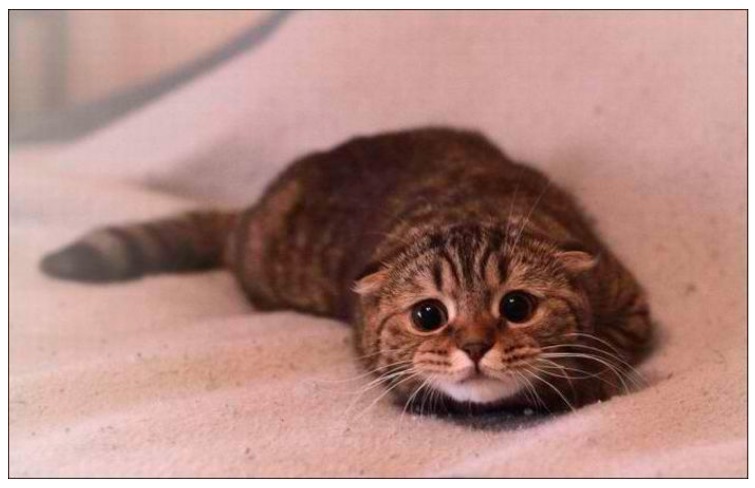
Frightened cat. Ears lowered, body lowered, dilated pupils, staring. Source: http://wrsouthshore.com/wp-content/uploads/2014/03/scardy-cat.jpg.

**Figure 3:**
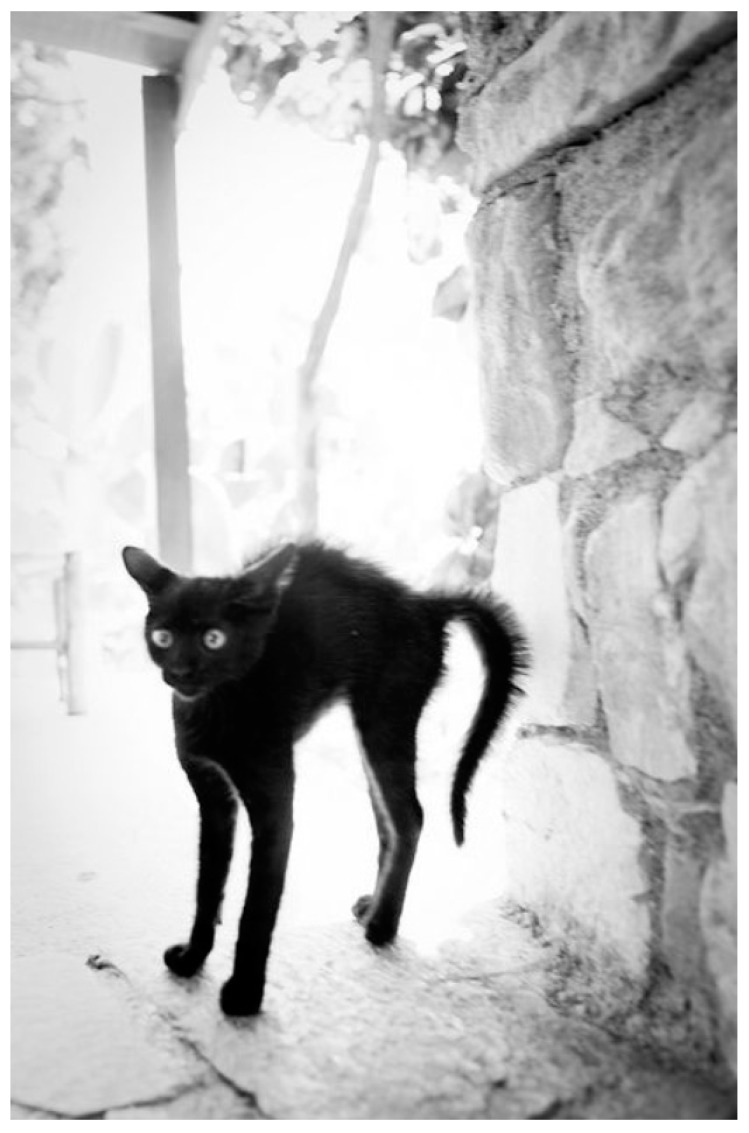
The “Halloween” cat. “Tippy-toes”, straight tail, arched back. Source: https://s-media-cache-ak0.pinimg.com/236x/77/e7/2b/77e72bd52e264155a346c00c986b6fa8.jpg.

Similar to dogs, fearful cats are tense and make themselves appear smaller by lowering their heads and leaning back (Figure 2). Signs of stress in cats also includes looking sleepy and exhibiting hypervigilance—although cats tend to move the direction of their ears rather than their heads. Agitated cats twitch their tails, and may act defensively if scared. Defensive cats appear to become even smaller, might hug a wall if available, move their ears down/back, and may hiss and react aggressively if unable to run away. Some cats appear to look bigger when alarmed and adopt the classic “Halloween cat” posture of standing on tippy-toes, with a straight tail (down or up) and arched back (Figure 3). The accompaniment piloerection (involuntary erection or bristling of hairs) in reactive cats is a physiologic response to stimulation of the sympathetic nervous system, and looking bigger is a secondary consequence of this reflex. The consequences of looking bigger is understudied, but the profile of a reactive cat should and can be recognised by humans (Kendall and Ley, 2006 [25]) and other animals. It is important for veterinary staff to be aware that a cat rolling on to its side when under duress is more likely preparing to defend itself than requesting a belly rub. The AAFP (American Association of Feline Practitioners) and the ISFM (International Society of Feline Medicine) feline-friendly handling guidelines (Rodan et al., 2011 [16]) illustrate the progressive signs of fear and anxiety in cats from early to late stages.

**Figure 4:**
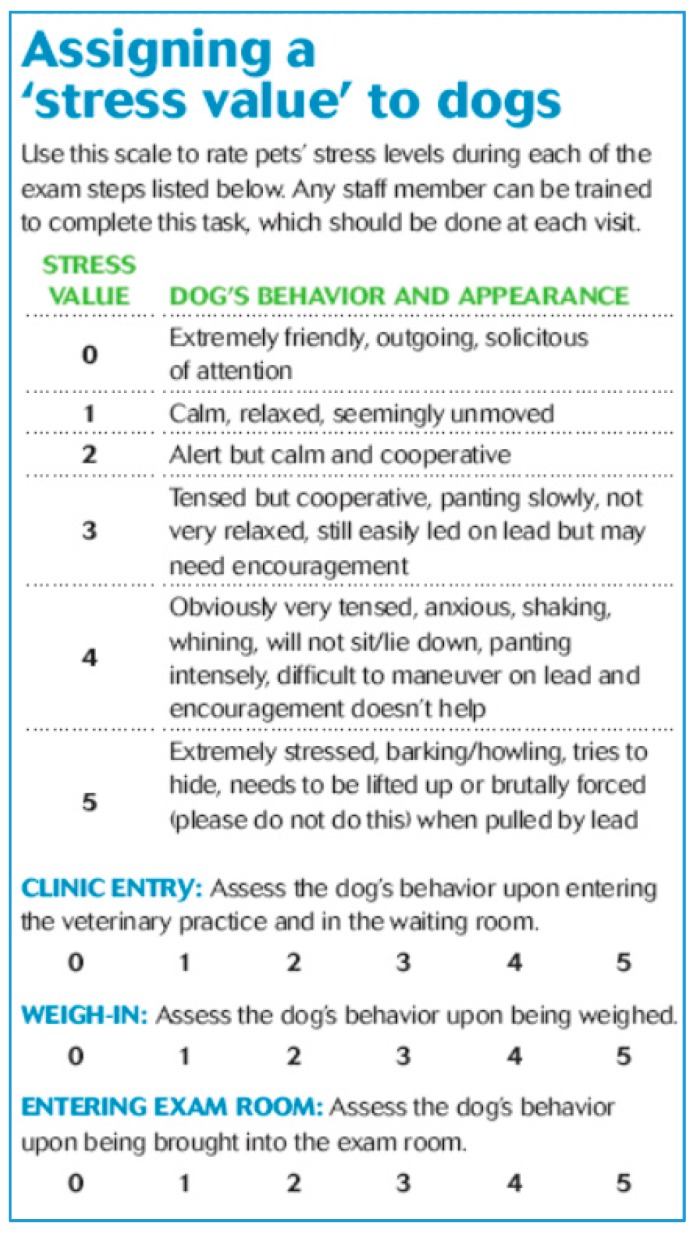
Clinic dog stress scale, from zero to five, on entry to the clinic, weigh-in and entering the exam room. Source: Overall, 2013 [5]. (Reprinted with Permission from dvm360, Oct. 2013, Facing fear HEAD ON by Karen Overal, M.A., VMD, PhD, DACVB, CAAB. dvm360 is published by Advanstar Communications, Inc. dba UBM.)

Useful protocols to help veterinary staff quickly assess the level of pain and stress in dogs and cats, and create a more “behaviour-centred” practice can be found in the Manual of Clinical Behavioral Medicine for Dogs and Cats (Overall, 2013 [23]) and accompanying DVD. Figure 4 shows the Clinic Dog Stress Scale, from 0 to 5, on: entry to the clinic; weigh-in; and entering the exam room (Overall, 2013 [5]). Dogs with zero scores are calm and dogs with a score of 5 are distressed and need help. Other scales available for assigning a stress value to dogs include: examination; blood sampling; diagnostic radiological procedures; and nail trimming (Overall, 2013 [23] (pp. 760–763)). By recording the animals’ responses at each visit, veterinary staff (and clients) can measure changes in the animals’ behaviour to help maximise patient care, as well as reducing the levels of relinquishment/euthanasia of problem dogs and cats. If the patient is extremely distressed and the exam is not medically urgent, it may be better to delay further evaluation and discuss anti-anxiety medications and behaviour modification with the client before the next visit. Working with the help of a legitimate trainer who uses “positive” training methods is likely to achieve the best outcomes. A handout, published by the American Veterinary Society of Animal Behavior (AVSAB) on choosing a trainer can be accessed from https://avsab.org/wp-content/uploads/2016/08/How_to_Choose_a_Trainer_AVSAB.pdf.

## 4. Preparation for Visits to the Hospital

If possible, preparation of and for the patient before its visit will help to prevent anxiety accumulating and alleviate stress on arrival at the hospital (Yin, 2009 [3]). Dogs and cats quickly learn to associate frightening or painful experiences with the hospital and staff through classical conditioning. The Bayer veterinary care usage study (Volk et al., 2011 [12]) indicated that many cats hid when the cat carrier appeared, became aggressive when put in the carrier, and cried during the journey to the veterinary hospital. This undesirable association can be lessened by classically conditioning a different association (classical counter-conditioning). For example, a fearful cat or dog can be trained to associate the “veterinary experience” including the carrier and the journey to the hospital with something pleasurable such as food. This approach could also be useful for patients presenting as emergencies, as training could be done pre-emptively, which may decrease stress during a subsequent emergency visit. The AAFP and the ISFM feline-friendly handling guidelines (Rodan et al., 2011 [16]) can help prepare clients and cats from the journey to the hospital to the journey home. Practical steps on crate training dogs and cats, and training for car rides can be found in Yin (2009 [3], Chapter 6) or on-line (Yin, n.d. [26]), and are also illustrated in Rodan et al. (2011 [16]). It would also behove pet owners to bring their pets to the hospital somewhat hungry, so that the pet is more likely to accept treats in the hospital setting. A favourite toy might also be efficacious in reducing stress.

## 5. Greeting Behaviour of the Veterinary Staff

Research shows that dogs are very good at reading and responding to signals of human intention (e.g., pointing) (Kirchhofer, Zimmermann, Kaminski, and Tomasello, 2012 [27]) and have large vocabularies of human words (Pilley and Reid, 2011 [28]). However, although humans recognise extreme states of stress and distress in dogs, they tend to misread more subtle signs (Mariti et al., 2012 [29]). In addition to learning what dogs and cats are trying to communicate to humans through movements of their eyes, ears and whiskers, their weight shifts, and body postures, veterinary personnel can also use their own body language to show pets that one is not a threat. Firstly, to minimise stress, care should be taken when initially greeting these animals. Avoid leaning over or reaching for an animal, as this can be construed as menacing (Yin, 2009 [3], 2012 [30]). Similarly, avoid squatting down with one’s face close to the animal; rather, squat (or sit) from further away and avoid facing head on. Squatting, sitting or standing sideways is less threatening than looming over an animal. Very fearful dogs can be approached by the person moving sideways before squatting down or sitting on a chair and turning obliquely. Direct eye contact should also be avoided (use peripheral vision). It is also important to allow the animal to gradually get used to personnel, even after a successful greeting. Slow, smooth movements, allowing the animal the opportunity to move away, and awareness of the animal’s body language will help to minimise stress.

A free poster on how to greet a dog can be downloaded from http://info.drsophiayin.com/greeting-poster. Useful information on preparing to greet a cat and interacting with the cat in the veterinary environment (including opening the carrier to access the cat) can be found in the AAFP and ISFM feline-friendly handling guidelines (Rodan et al., 2011 [16]).

## 6. Handling and Restraint

Veterinary staff should be aware of how their interactions affect the patients, and learn to choose the best method of control while working in a calm and positive manner. Over-restraining an animal is frightening and painful. Knowing how to provide adequate restraint, support and direction to the animal will help it feel balanced and safer. For this reason, it is better to disassemble carriers for cats and small dogs that are reluctant to exit the carrier rather than dumping the animal out or grabbing it by the scruff (Yin, 2009 [3]; 2012 [30]).

Once the pet has been greeted appropriately, it should be handled in a manner that helps it to know what the handler wants rather than confusing it. Pets should be guided into the appropriate position rather than flipped in a rough manner, which may engender distrust (Yin, 2009 [3]; 2012 [30]). The minimal amount of restraint that is necessary for immobilisation should be used. Examine patients where they are most comfortable; cats and small dogs may feel more comfortable being examined on the veterinarian’s lap. Cats often prefer being examined in a structure with sides (e.g., on weighing scales or in the bottom half of a carrier) and can be partially hidden under a blanket. Many dogs are more comfortable being examined on the floor, rather than on the exam table (Yin, 2009 [3]; 2012 [30]); or in some difficult cases, outdoors in a secure environment.

Excellent resources on low stress handling, restraint and behaviour modification of dogs and cats in the veterinary environment, including moving patients around the hospital, have been produced by the late Sophia Yin (Yin, 2009 [3]; 2012 [30]), much of which can be freely accessed on-line at https://drsophiayin.com/. In addition, veterinary hospitals and/or individuals can become accredited in Low Stress Handling™ through Yin’s online or DVD training sessions and testing at http://216.243.143.215/lowstress/certification. Training enables staff to hold, examine and treat dogs and cats in ways that ensure the patients are as comfortable as possible.

When pets exhibit fear and anxiety, the response of the veterinary staff (and owner) can either further aggravate or lessen the pet's fear. It is important not to punish an animal that is fearful. Physical punishment or even a raised voice can increase fear and elicit aggression. Dogs that have been punished for growling, or rewarded by (the necessary) retreat of staff may learn to bite without warning (i.e., through negative reinforcement). Management products such as head halters and muzzles, and pharmacological intervention may be required for the safety of people; medication may also enhance the pet’s emotional wellbeing (Landsberg, 2009 [31]). Knowing which body part to pet a dog and cat also matters. A study by Kuhne, Hößler, and Struwe (2014 [32]) showed that being petted around the head, neck and muzzle or being held at the collar was perceived as unpleasant by dogs. Thus, dogs manipulated in such regions may feel more entrapped, which may affect their behaviour choices. Cats appeared to prefer being stroked the most in the temporal region (between the eyes and ears), followed equally by the perioral area (chin and lips) and other areas where cats did not have glands, and least in the caudal region (around the tail) (Soemichsen and Chamove, 2015 [33]).

Further tips to reduce stress during procedures include the use of butterfly catheters, where appropriate, to make taking blood samples easier, and using a vacutainer-type adapter to fill the tubes directly (thus eliminating a step) and reducing the risk of venous collapse associated with syringes. Topical local anaesthetic creams or gels may also reduce the discomfort of venepuncture and should not be over-looked when taking temperatures and emptying anal sacs (Overall, 2014 [34]).

## 7. Medications for Behavioural Management

When using medication for behavioural management, it is best to deliver intravenous drugs before the patient is hyperaroused and to use an appropriate anxiolytic. Clinicians may medicate/sedate, for example, an extremely fearful dog in the car prior to it entering the veterinary hospital. If the dog or cat had a stressful experience en route to- and/or at the veterinary hospital, administering anti-anxiety medication during the appointment may cause the animal not to complete the full neuromolecular process of making memories of the experience (Overall, 2014 [34]). This may help the animals not to loathe visits to the vet by association. Overall, (2014 [34]) also advocates that the brains of distressed dogs may be buffered against damage caused by the stress by administering 1,200 to 1,500 mg of omega-3s daily.

It should be noted that the drug acepromazine (Ace, ACP) is contraindicated for treating anxious (Frank, Gauthier, and Bergeron, 2006 [35]) or aggressive animals as they are more reactive to noise and startle under its influence (Overall, 1997 [36]), and can produce marked sedation (Thompson, 1998 [37]). Acepromazine is a phenothiazine derivative tranquiliser that works by dissociative effects. This means that an animal might still perceive the fearful stimulus but be unable to make sense of it cognitively, resulting in increased fear and atypical reactions (Overall, 1997 [36]). Phenothiazines have also been shown not to be efficacious at reducing stress during transport in dogs (Bergeron et al., 2002 [38]; Frank et al. (2006 [35]). Frank et al. (2006 [35]) suggests that clomipramine (a tri-cyclic antidepressant (TCA) currently labelled in several countries for the treatment of separation anxiety and obsessive-compulsive disorders in dogs) might be a better choice for transport and other stress, as it decreased behavioural signs (moving and panting) and physiological measures (plasma cortisol, heart rate) associated with fear and anxiety in dogs. Other anxiolytic agents, such as benzodiazepines (BZs), can be administered on an as-needed basis along with the on-going use of a TCA for a visit to the veterinary hospital and other stress-evoking events such as thunder storms and owner departures Landsberg et al. (2013 [18]). Note: Cats, and to a lesser extent dogs, can have a rare paradoxical excitement response to BZs (Overall, 2007 [39]). Frank et al. (2006 [35]) study also mentions that, anecdotally, some veterinarians report using clomipramine or selective serotonin reuptake inhibitors (SSRIs), such as fluoxetine or fluvoxamine for travelling dogs and cats. However, Landsberg et al. (2013 [18]) states that while TCAs may be used in pets for the same issues as SSRIs, TCAs (especially those that sedate) may be more effective for calming, and an SSRI such as fluoxetine may be more likely to cause agitation. It should be noted that while clomipramine is sometimes prescribed for use in cats, caution must be exercised regarding its use. TCAs are metabolised through glucuronidation—a pathway for which cats have decreased facility compared to dogs, and hence cats might be more sensitive to this class of drug (Lainesse, Frank, Beaudry, and Doucet, 2007 [40]; Overall, 2013 [23]). It is not unusual to see drowsiness or an increase in the effects of other medications when a dog or cat has been given medication for behavioural management.

Although this article focuses on reducing stress in the veterinary hospital or clinic, many animals are chronically stressed or suffer from anxiety, and might benefit from an ongoing care plan combining behaviour/environmental modification and behavioural drug therapy. “Washout periods” for animals that have taken a monoamine oxidase inhibitor (MAOI) such as selegilene or amitraz must be in place before being prescribed TCAs, SSRIs or with any medication or dietary supplement affecting serotonin (including but not limited to amitraz (Landsberg et al., 2013 [18]; Overall, 2013 [23]) because of risk of serotonin syndrome (Box 1). Laboratory evaluations might be indicated prior to administration of medications for behavioural management especially in older or compromised animals.

Box 1 **Serotonin syndrome.**

Serotonin syndrome, or serotonin toxicity, is a dangerously high level of serotonin causing mainly neurological, autonomic and gastrointestinal signs, and can be fatal if untreated (Landsberg et al., 2013 [18]). Signs include agitation, mental confusion, hyperaesthesia, shivering, shaking, hyperthermia, tachycardia, tachypnoea, abdominal pain, diarrhoea, vomiting, hypersalivation, tremors, seizures, coma and death (Gwaltney-Brant, Albretsen, and Khan, 2002 [41]). Animals that have been exposed to acaricides and tickicides containing the MAOI amitraz (including washes, spot-ons and collars) in the last 14 days or so should also not be given serotonin enhancing medications. Note: Amitraz is not licensed for use in cats and accidental contact with dog products containing this active ingredient is potentially serious. Other products that have the potential to induce toxic levels of serotonin include, but are not limited to, tramadol (analgesic) and herbal supplements that the owner may use such as St John’s wort (Crowell-Davies and Landsberg, 2012 [42]).

There are many excellent resources available concerning appropriate medications to use when treating fearful and anxious patients including routes of administration, dosages, polypharmacy, wash-out periods etc. The following textbooks are good resources to consult on these matters: Horwitz and Mills (2012 [43]), Landsberg et al. (2013 [18]), and Overall (2013 [23]). A guide to understanding how behavioural medications work by Overall (2007 [39]) can be accessed on-line, as can a handout on helping practitioners find the right medications to prevent and alleviate fear and distress in dogs and cats (including helping them not make fearful memories), and medications to quell nausea for travel (Overall, 2014 [44]). The AAHA (American Animal Hospital Association) has produced behaviour management guidelines to help veterinary staff ensure that the basic behaviour needs of canine and feline patients are understood and met in practice (Hammerle et al., 2015 [17]), including pharmacological intervention. A valuable source of information on psychoactive herbs used in veterinary behaviour medicine has been produced by Schwartz (2005 [45]).

## 8. Creating a Pet-Friendly Environment: Hospital Design

The design of the hospital is very important to put patients and clients at ease. Research has shown that cats in shelters become highly distressed when they saw or heard dogs (McCobb, Patronnek, Marder, Dinnage, and Stone, 2005 [46]), probably because the cats had no way of escaping from the dogs and no means of hiding within their cages. Thus, it is important that feline contact with dogs be minimised within the veterinary environment (Lloyd, 2008 [47]). A student report (Hernander, 2008 [48]) found that dogs that had recently visited the veterinary clinic had higher levels of stress than those that had not visited recently. These findings have profound implications for the treatment provided by veterinary staff, as perceived by the dogs and cats.

### 8.1. The Waiting Room

Hernander (2008 [48]) reported that dogs that had waited in waiting rooms that were not chaotic, and had sufficient time to calm down were less stressed than dogs that were moved quickly. The Bayer veterinary care usage study (Volk et al., 2011 [12]) showed that cats also displayed signs of stress and fear in the veterinary clinic waiting room, particularly when unfamiliar dogs were present. Yin (2009 [3]) advocates setting up the hospital so that the first thing the pet sees on arrival is a reception desk and not other animals. Visual barriers in the waiting rooms provide species-specific areas, and cat carriers can be placed in raised and enclosed areas. Tasty treats should be placed in strategic positions such as the reception desk or near the weighing scale.

Hernander’s (2008 [48]) study discovered that dogs found being weighed (on scales) to be more stressful than sitting in the waiting room. Fear of being weighed may be reduced by allowing the dogs more control over their participation in the process. Dogs that are not afforded some measure of choice have less chance to develop self-control and autonomy (Lloyd and Roe, 2012 [49]), and may be more reactive in the veterinary environment. Thus, dogs could be trained to not fear being weighed by being rewarded for making desirable behaviour choices (positive reinforcement), and by placing the scales in the floor area, rather than in corners where dogs may be more reluctant to go. Dogs have dichromatic colour vision (further discussed below) and can discern the colour blue (Neitz, Geist, and Jacobs, 1989 [50]), hence it may be beneficial to paint floor scales in this colour to demarcate it from the surrounding floor area. Cats should be weighed in the exam room, and can be weighed in the crate (carrier) (with the weight of the crate/towel being subtracted later). An underused technique described by Overall (2014 [34]) is for cats that have already been clicker-trained to jump onto and stand on a scale—a technique that is easiest to use with bold cats/kittens and one that the clients enjoy. Client comfort should also be kept in mind, as relaxed owners help to keep their pets calm. The use of synthetic pheromones, and other calming agents, to reduce stress are discussed below.

### 8.2. The Exam Room

Exam rooms should be made as inviting as possible by having comfortable chairs for clients and soft, non-slip rugs/towels for pets. A variety of tasty treats and toys (washable) should also be available. Giving animals time to habituate to the environment, as described above (Hernander, 2008 [48]) can go a long way to reduce stress. Along with witnessing staff who project caring attitudes, pet-friendly acoustic and other sensory environments (described below) are also likely to appeal to the client, who may feel better about leaving his/her pet in the hospital.

### 8.3. Cages and Housing

**Figure 5:**
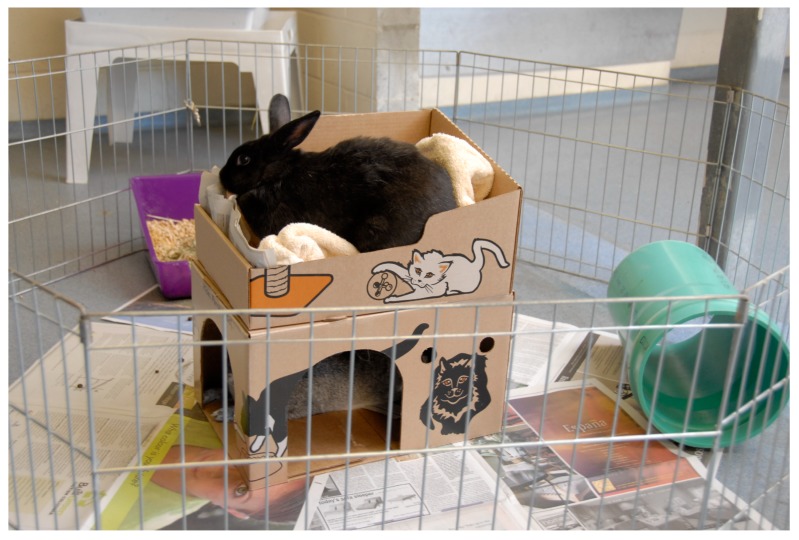
The Hide, Perch & Go™ box providing hiding, perching and scent-marking opportunities for a rabbit. Reproduced with kind permission from the British Columbia Society for the Protection of Cruelty to Animals, Vancouver, Canada.

Kennels and treatment areas (discussed below) are potentially areas that cause high stress. Cats should be housed separately from dogs, and both should be kept in kennels that face walls rather than other animals to decrease visual contact. The natural instinct of a cat when exposed to a threatening situation is to retreat, however if this is not possible then it will attempt to conceal itself from view (Kry and Casey, 2007 [51]). Providing cats with hiding places, such as a cardboard box (that they can also use as an elevated platform should they choose) allows cats to perform this coping behaviour, possibly affording a sense of control/autonomy over the environment, and alleviates stress (Kry and Casey, 2007 [51]). The simple act of partially draping a towel over the cage door makes cats less exposed, thus reducing stress. Indeed, some cats will not eat or use their litter tray in full view of veterinary staff. In Canada, the British Columbia SPCA has developed the Hide, Perch & Go™ box to help reduce stress in shelter cats. The box is made of plasticised cardboard and enables the cat to exhibit natural behaviours of perching on top in a shallow tray or hiding inside. The box also serves as a cat carrier; hence the owner can take a newly adopted cat home in a familiar and scent-stable environment. The box has been adapted for use in veterinary hospitals and isolation areas where cats may spend a large amount of time in a potentially stressful environment (Lloyd, 2008 [47]). While there is no supporting scientific evidence, the box also appears to be beneficial for rabbits by providing hiding, perching and scent-marking opportunities (Figure 5). Dogs and cats, and other animals, could be given the option to spend quality time working for their food, for example, by eating from a stuffed KONG toy to alleviate boredom. This type of toy can be filled in advance with wet food (or, where appropriate, a small amount of peanut butter or processed cheese sauce as a treat) and frozen for later use; frozen contents take longer to eat, thus enhancing the environmental enrichment.

### 8.4. The Acoustic and Olfactory Environment

Areas that are often overlooked regarding hospital design that may increase stress are how to combat noise and odour. Although the responses of dogs to music has been studied fairly extensively, the effects of the acoustic environment on canine and feline stress (and staff) levels in the veterinary clinic is lacking. Soothing music (and possibly even TV) might benefit some animals. One study (Kogan, Schoenfeld-Tacher, and Simon, 2012 [52]) found that playing “classical music” increased the amount of time kennelled dogs spent sleeping and decreased vocalising compared to other music or no music, and “heavy metal” music increased body shaking (or trembling). In another study of kennelled dogs, Wells, Graham, and Hepper (2002 [53]) also found increases in resting postures and decreased barking to classical music, while heavy metal music elicited increased barking. However, research on the effects of sound in dogs is unclear, and it may be that music is not the most effective method of acoustic enrichment, for dogs at least. A recent study by Brayley and Montrose (2016 [54]) found that the reading of audiobooks was more effective in enhancing the welfare of kennelled dogs than other auditory conditions including classical music. The authors concluded that as dogs are highly social animals whose welfare is enhanced by human interactions, audiobooks may somewhat fulfil this role and provide the illusion of company and comfort.

McConnell (2013 [55]) purports that it is not the classification of the music that matters, but the features of each piece that make the difference. Classical music includes a broad range of pieces, from soothing selections to the more raucous, and McConnell (1990 [56]) found that short, rapidly-repeated notes increased motor activity in dogs, and that long, continuous notes decreased activity. Although it is not certain what are the best acoustic features to calm dogs in an over-stimulating environment, some kinds of music or other sounds seem to have a positive effect on kennelled dogs, especially sounds with long, extended notes, pure tones and relatively slow tempos. A study on cats (Snowden, Teie, and Savage, 2015 [57]) found that for auditory enrichment to be effective, it must contain features relevant to the target species. This study suggests that cats find music that is “species-specific” to be more appealing than music made for humans. Cats vocalise one octave higher than people, and in Snowden et al. (2015 [57]) study, cats preferred music with a higher pitch and a tempo based on purring and the suckling sound made during nursing. As for any enrichment, the goals must be considered. It is not sufficient to simply turn on a radio or play some classical music and assume that acoustic enrichment needs are being met—if the goal is to calm an agitated animal then melodic music based on affiliative/rewarding sounds is likely to be more effective than short note durations and discord (Snowden et al., 2015 [57]).

Sick animals may need to be kept in the treatment area in order to be monitored, but noise levels and movement should be kept to a minimum. Noise levels can be minimised by the use of quiet clippers, nonslip matters on tables to reduce noise and the use of one-way mirrors for monitoring patients. With regards to hospital design, Moser (2004 [58]) reported that sound can be absorbed by installing products with high noise-reduction coefficient ratings such as sound-absorbing acoustic ceiling tiles, baffles, and wall panels. Solid doors absorb more sound that hollow-core doors, and high ceilings minimise sound bouncing off them. Moser (2004 [58]) also suggested that odours can be minimised by good hygiene, good drainage, and good ventilation.

Dogs and cats obtain a lot of information through olfaction, and scent mark with urine and faeces as well as with facial and body secretions (Overall, 2014 [34]). Animals that are exquisitely sensitive to odours are likely to find the olfactory environment of a veterinary hospital stressful. A solution of bleach, even as low as 1%, can destroy olfactory neurones (Overall, 2013 [23]) resulting in a loss of information and heightening anxiety. Airing out rooms and using disinfectants that do not have discernibly strong odours followed by air-drying may help minimise this stress. Overall, 2014 [34] suggests that because cats (and dogs) are intrigued by odorant molecules in scent trails, opening a window (where appropriate) may result in the animals paying more attention to these odours than to stressors.

### 8.5. Colour and Lighting

It may behove veterinarians to paint or decorate their practice in colours that are positively perceived by dogs and cats. Architect Heather Lewis is currently working on what colours and lighting might be calming for dogs and cats in the veterinary environment. Dogs have dichromatic vision, see better in low light than humans can, and can see into the ultraviolet UVB spectrum. Cats also see into the UVB spectrum and in low-light situations, and although they are not believed to see colours with as much precision as humans do, they have trichromatic vision (Lewis, 2015 [59]). The ability to see the UVB spectrum means that some materials are likely to appear fluorescent to dogs and cats, including organic material such as urine that contains phosphorous. Bright white, man-made materials such as paper, plastic and white fabrics are likely to be visually jarring—“Your professional-looking white doctor’s coat could be lighting up like a Christmas tree to your patients” (Lewis, 2015 [59]). Lewis (2015 [59]) suggests that “fear free” colours for dogs and cats include hues in the soft yellow to violet range, avoiding oranges and reds, and dark colours. Because dogs and cats do not see well in low light, using lighter colours in darker areas are likely to make them feel more at ease, as dark colours make it harder to see and comprehend their surroundings. A “visually comfortable” colour palate for dogs and cats can be found on-line (Lewis, 2015 [59]).

## 9. Creating a Pet-Friendly Environment: Other Calming Agents and Techniques

In addition to affording animals more control over their environment and consideration of hospital design, other measures can be used to alleviate stress and anxiety. The use of pheromones and techniques that utilise pressure touch, and aromatherapy may be useful to decrease arousal in the veterinary setting.

### 9.1. Pheromonotherapy

Pheromones are a type of chemical communication used by members of the same species. Pheromonotherapy is the use of synthetic pheromones such as Feliway^®^ for cats and Adaptil^®^ (dog appeasing pheromone (DAP)) for dogs to reduce anxiety and to facilitate adaptation to unfamiliar environments. Studies have been conducted to investigate the efficacy of these products to lessen the distress of dogs and cats in the clinical environment and other settings. However, with the exception of one study that indicted DAP reduced fear or anxiety of puppies during training resulting in better socialisation (Denenberg and Landsberg, 2008 [60]), a systematic review of the scientific literature by Frank, Beauchamp, and Palestrini (2010 [61]) found insufficient evidence of the effectiveness of pheromonal products for treating undesirable behaviours.

The limitations in the quality of the evidence does not mean that pheromonotherapy does not work, and although further research is warranted, it is used broadly in veterinary medicine. These products can be sprayed on bedding, inside cages/crates or directly on veterinary personnel, or used as diffusers. No adverse effects have been reported in dogs and cats (Crowell-Davies and Landsberg, 2012 [42]) (and the author found no recent studies to indicate otherwise), but care should be taken when using pheromone diffusers or sprays around birds and fish, due to their sensitive respiratory tracts. Pheromone products are also used to help calm animals at home and en route to the veterinary hospital.

### 9.2. Pressure Touch

TTouch (bodywork) is used to reduce stress and relax animals, and may play a role in sensory-enhanced learning (Lloyd and Roe, 2014 [62]). The bodywork aspect of TTouch consists of specific touches and body wraps. Touches are based on precise circular movements, slides and lifts that can be applied all over the body to help an animal relax and identify areas of tension. Body wraps are useful for calming excitable or fearful animals. The following excerpt from Lloyd and Roe (2014 [62], (p. 11)) explains how touch may help an animal relax:

Touch has been reported to have calming effects in a variety of animals and people including children with autistic disorders, premature babies (swaddled), normal babies and adults (Grandin, 1992, 2007). An overview of Touch triggers a raft of changes in the nervous system, from local chemical responses to the release of endogenous opioids. The effect of touch in stimulating the skin has a massive, indirect effect on the rest of the body (Lindley, 2009), including the release of oxytocin and prolactin (amongst other hormones) which are important for bonding and nurturing (Case, 2005; Odendaal and Meintjes, 2003). Physical contact, such as stroking, has also been found to induce physiological relaxation of the heart in pet dogs (McGreevy, Righetti and Thomson, 2005).

**Figure 6:**
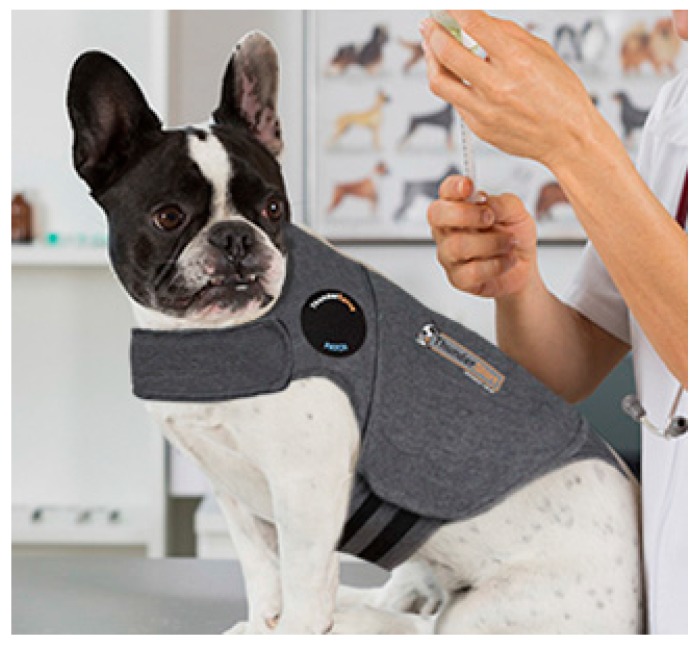
The ThunderShirt. A compression garment used to help dogs (and cats) feel more secure, based on the concept of swaddling. Source: http://www.thundershirt.com/media/review/TWVet-SS.jpg.

**Figure 7:**
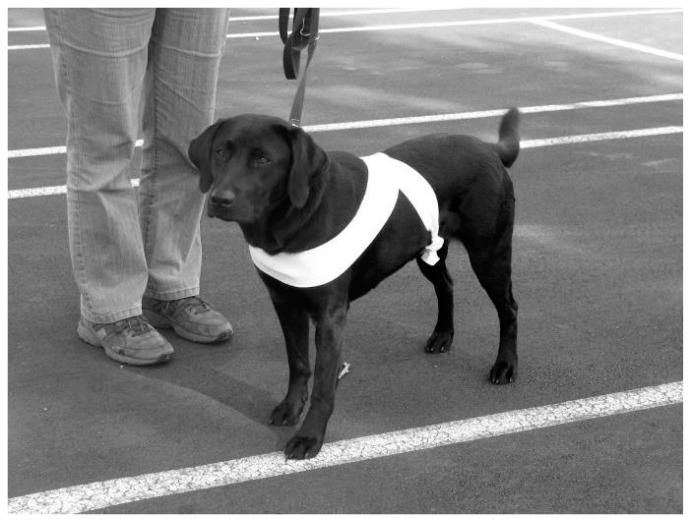
Application of an elastic bandage in a figure of eight (half wrap). The inherent stretch in the fabric provides a light sensation of localised pressure against the body, and is used with the intent of calming excitable or fearful dogs. Photo courtesy of Lloyd and Roe (2014 [62]).

**Figure 8:**
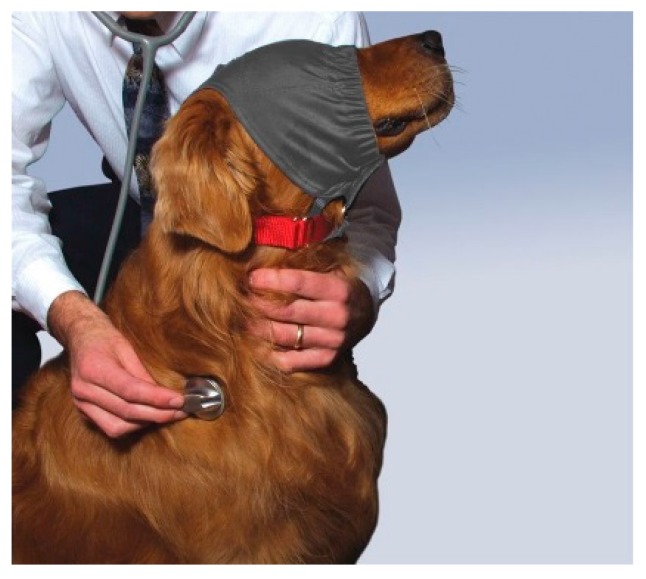
The Calming Cap. Filters the dog’s vision to reduce the visual stimulation that the dog experiences to help in stressful situations. Source: http://www.thundershirt.com.au/media/catalog/product/cache/2/image/1200x/040ec09b1e35df139433887a97daa66f/c/a/cap_2.jpg.

A thunder jacket, a compression garment that applies pressure (such as the ThunderShirt), can be used to help dogs (Figure 6) and cats feel more secure. However, a simple and effective wrap can be made from an elastic bandage (Figure 7) to create localised pressure, based on the concept of swaddling. Another useful product that may ease canine anxiety in a high-stress situation is the Calming Cap (Figure 8). The cap is made from a sheer fabric that reduces visual stimuli that may worry a dog, while allowing the dog to manoeuver through the environment; it may also be useful to calm the dog on the car journey to the veterinary hospital.

### 9.3. Aromatherapy and Other Olfactory Stimuli

Aromatherapy is the practice of using volatile plant oils, including essential oils, for psychological and physical well-being. Graham, Wells, and Hepper (2005 [63]) suggest placing lavender or chamomile essential oil on bedding to help dogs relax. This study also found that rosemary and peppermint oil encouraged standing, moving and vocalising, and so should be avoided in this setting. Essential oils are probably best avoided in feline environments. Cats lack the enzyme UDP-glucuronosyl transferase, which renders the cat susceptible to the potential toxic effects of some essential oils (as well as phenol-based disinfectants) (Addie et al. 2015 [64]). Because cats spend a significant amount of time grooming, they may also orally ingest oils and other substances they come into contact with in their environment. Note: Essential oil toxicity has also been reported in dogs. Genovese, McLean, and Khan (2012 [65]) noted that dogs (and cats) can experience significant adverse effects when exposed to flea preventatives derived from plants (most commonly, agitation and hypersalivation in cats, and lethargy and vomiting in dogs), so caution must be exercised whenever essential oils are being used.

Although a wide variety of feline species have been shown to gain welfare benefits from the introduction of olfactory stimuli to the captive environment, the effect of this stimulation on the domestic cat has been largely overlooked. However, one study (Ellis and Wells, 2010 [66]) that explored the influence of olfactory stimulation on cats housed in a rescue shelter determined that certain components of the cats’ behavioural repertoire were influenced by olfactory stimulation. Catnip and prey scent (rabbit) encouraged a significantly higher frequency of behaviours indicative of reduced activity (e.g., more time sleeping, less time standing and actively exploring the environment) in comparison to the control condition. Catnip also encouraged play-like behaviour characterised as the “catnip response”, which may hold potential as environmental enrichment for hospitalised cats.

### 9.4. Other Complimentary Therapies

Although often viewed with suspicion, other complementary therapies, such as Bach flowers (tiny amounts of flower essence used to calm emotional animals), Reiki and Shen (the channelling of “energy” by the therapist into the patient) are employed in some veterinary practices to treat a range of emotional issues in animals (Lindley, 2012 [67]). There are courses available in the techniques described for veterinary staff to become accredited complementary therapy practitioners, however, it is important to remember that the use of these techniques is not well supported in the scientific literature, and that behaviour therapy should be continued alongside any other intervention.

## 10. Conclusions

Minimising stress must be a standard of care. Continual stress in the veterinary environment is undesirable for welfare reasons, as well as for adverse effects on immune function, rate of recovery, and increased risk of injury to staff. Benefits of creating pet-friendly, low stress experiences are numerous. By identifying a puppy or kitten that is fearful or anxious early in life, and minimising the role of veterinary care in inducing and maintaining fear, staff can positively affect the patients’ well-being. Client confidence is improved, as patients are more settled and staff project a caring attitude. Staff increase their skills in handling animals, resulting in better job satisfaction and fewer bites and scratches. The hospital also benefits by clients being more likely to bring their pets back, and increased staff efficiency due to less time spent on restraining animals. Last but by no means least, the patients are likely to be more relaxed and compliant on their next visit.
